# The Efficacy of Adjuvant Chemoradiotherapy in Early-Stage Gallbladder Adenocarcinoma Depends on the Tumor Invasion Depth and Differentiation Level

**DOI:** 10.3389/fonc.2020.616170

**Published:** 2020-12-18

**Authors:** Hui Liang, Yifan Wang, Jie Chen, Jiajun Xing, Yabin Pu

**Affiliations:** ^1^ Department of General Surgery, Naval Medical Center of PLA, Shanghai, China; ^2^ Department of Cardiothoracic Surgery, Naval Medical Center of PLA, Shanghai, China; ^3^ Department of Orthopedics, Naval Medical Center of PLA, Shanghai, China

**Keywords:** gallbladder adenocarcinoma, adjuvant therapy, differentiation, invasion, survival

## Abstract

**Background:**

Although the performance of adjuvant chemoradiotherapy (ACRT) for resected gallbladder cancer may improve the survival for certain patients, its impact on the survival in early-stage resected gallbladder adenocarcinoma (GBAC) patients remains underexplored. This study aimed to determine the ACRT effects on the survival of early-stage resected GBAC patients.

**Methods:**

Patients with early-stage resected GBAC diagnosed between 2010 and 2016 were selected from the Surveillance, Epidemiology, and End Results (SEER) database. The covariables included gender, age, race, tumor differentiation, TNM stage (AJCC TNM staging system, 7^th^ edition), adjuvant radiotherapy (ART), and adjuvant chemotherapy (ACT). The effects of ACRT on survival were evaluated by univariate and multivariate analysis.

**Results:**

A total of 1,586 patients with resected GBAC met the inclusion criteria were included in this study. Patients who received ACT were older, with poorer tumor differentiation or higher TNM stage (all p < 0.05), while patients who underwent ART were proved to be significantly correlated with poorer tumor differentiation (p = 0.010) and higher TNM stage (p < 0.001). Univariate and multivariate analysis of overall survival (OS) showed that age (p < 0.001; HR, 2.039; 95% CI, 1.718–2.420), tumor grade (p < 0.001; HR, 1.887; 95% CI, 1.530–2.370), and AJCC 7th TNM stage (p < 0.001; HR, 1.417; 95% CI, 1.182–1.699) were independent prognostic risk factors. Interestingly, ART and ACT were not independently associated with improved OS in the overall cohort analysis. However, when patients were subgrouped according to tumor differentiation, ART (p = 0.049; HR, 0.639; 95% CI, 0.409–0.999) has been identified as a significant prognostic factor for grade III/IV patients. Meanwhile, ARC (p = 0.011; HR, 0.739; 95% CI, 0.586–0.932) was associated with improved OS among tumor stage II patients (p<0.001).

**Conclusion:**

ACRT may have specific survival benefits for early-stage resected GBAC patients. ART can improve survival in patients with poor or absent tumor differentiation. Besides, patients with tumor invasion beyond muscularis (stage II tumor) may benefit from ACT. Our study provides supporting evidence for the clinical applications of ACRT in early-stage GBAC patients.

## Introduction

Gallbladder adenocarcinoma (GBAC) is the most common biliary tract system cancer, often presenting at an advanced stage at the time of the first diagnosis and with a poor prognosis due to its aggressiveness. The 5-year survival rate is <5% for patients with metastasis and <35% for those with locally advanced disease (1–5). Surgery remains the only potentially curative therapy for GBAC (2, 6). However, even after complete resection, many patients experience locoregional and/or distant recurrences (7–9). Thus, there is considerable interest in exploring the potential benefit of additional adjuvant treatments, such as adjuvant chemoradiotherapy (ACRT), especially for early-stage GBAC. Most published studies of adjuvant treatments for the GBAC have been obtained at a single medical center with few patients due to the rarity of this disease (10–16). Large-scale prospective clinical trials to are difficult to conduct, and consequently, clinicians have little evidence to determine whether adjuvant therapies would be beneficial for early-stage GBAC patients. In this scenario, evaluating the ACRT benefit in GBAC patients can clarify these important clinical issues.

Therefore, in this study, data retrieved from the Surveillance, Epidemiology, and End Results (SEER) database were used to evaluate ACRT impact on the survival of early-stage resected GBAC patients and provided evidence for the ACRT clinical application in these patients.

## Methods

### Data Resource

The analyzed data were obtained from the SEER*Stat public database (www.seer.cancer.gov), Rate Session: Incidence - SEER 18 Regs Custom Data (with additional treatment fields), Nov 2018 Sub (1975–2016 varying).

The inclusion criteria for this study were to be a GBAC patient resected in an early stage (stages I and II) with pathological diagnosis from 2010 to 2016. Patients receiving preoperative or unknown radiotherapy, or censored within one month after surgery, were excluded. Clinicopathological indicators, such as gender, age, race, tumor differentiation, AJCC 7th TNM stage, adjuvant radiotherapy (ART), chemotherapy (ACT), survival information, were recorded for analysis.

### Definition

According to the AJCC TNM staging system (7^th^ edition), stage I means T1N0M0, and stage II means T2N0M0. T1 is defined as the tumor invasion limited to the lamina propria and the muscularis, while T2 is defined as the perimuscular connective tissue invasion, but not exceeding the serosal layer or entering into liver. For survival analysis, overall survival (OS) was calculated by the time interval from diagnosis to death or censoring.

### Statistical Analysis

The SPSS software (version 20.0) was used for statistical analyses and the GraphPad Prism 8 software was applied to depict the survival curves. For the data presentation, the medians along with inter quartile ranges (IQRs) were used for continuous variables and frequencies with percentages for categorical variables. The Kaplan–Meier method with log-rank test was used to calculate and compare the median survival. To identify independent prognostic factors, univariable and multivariable Cox proportional hazards regression analyses were performed and further subgroup analyses were applied. P values < 0.05 were considered statistically significant.

## Results

### Clinicopathological Characteristics

**Figure 1:**
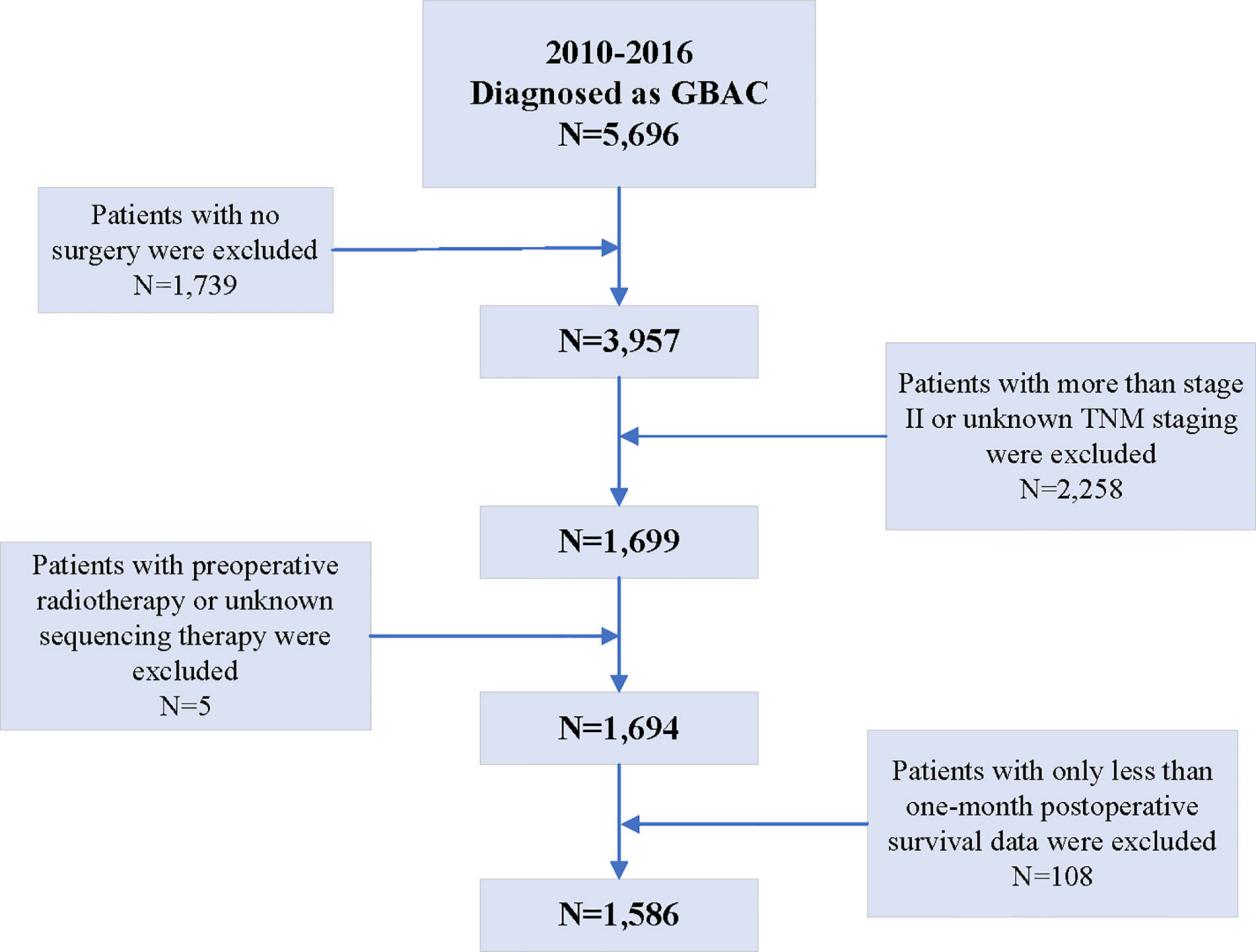
The flowchart of the inclusion and exclusion criteria in this study.

According to the inclusion criteria, among 7,729 patients diagnosed as gallbladder cancer between 2010 and 2016 present in the SEER database, 5,696 were confirmed as GBAC. Then, 1,739 patients were excluded because they did not undergo any surgery and another 2,258 patients were excluded because they were diagnosed with a TMN stage greater than II or unknown. Additionally, five patients undergoing preoperative radiotherapy or unknown sequencing therapy were excluded. Finally, 108 patients with postoperative survival data of less than 1 month were also excluded. Therefore, based on these criteria, 1,586 resected GBAC patients were finally included in the analyses of this study (**Figure 1**).

The median age of the patients was 72 (IQR 62–81) years and there were 1,091 (68.8%) female individuals in the studied cohort. The main ethnicity of patients was white (76.4%), and black and others (American Indian/AK Native, Asian/Pacific Islander) only accounted for 12.7 and 10.9% of patients, respectively. According to the AJCC TNM staging system (7^th^ edition), there were 525 (33.1%) patients with stage I and 1,061 (66.9%) with stage II. Regarding the tumor differentiation degree, 385 (24.3%), 579 (47.9%), and 338 (21.3%) patients separately owned grades I, II, and III+IV. Concerning the adjuvant therapy, 35 (2.2%) patients received only ART, 160 (10.1%) received only ACT, and 146 (9.2%) received both.

### The Relationships Between Adjuvant Chemotherapy and Clinicopathological Characteristics

Significant positive relationships were found between ACT and age (p < 0.001), tumor grade (p = 0.021), and AJCC 7th staging (p < 0.001) (**Table 1**). Older patients, with less tumor differentiation or with higher TNM stage were more likely to receive ACT. However, receiving ART showed to be significantly correlated with poorer tumor differentiation (p = 0.010) and higher TNM stage (p < 0.001) (**Table 2**). Thus, tumor differentiation and TNM stage may be the most popular evaluable indications for the enrollment of ACRT in current clinical practice.

**Table 1:** The relationships between adjuvant chemotherapy and clinicopathological characteristics.

Variables	No. of patients(n = 1,586)	Without ACT(n = 1,280)	Receiving ACT(n = 306)	P value
**Gender** Female Male	1,091 495	879 401	212 94	0.836
**Age** <70 >=70	673 913	489 791	184 122	**<0.001**
**Race** Black White Others	202 1,211 173	157 985 138	45 226 35	0.458
**Grade** I II III+IV Unknown	385 759 338 104	326 609 257 88	59 150 81 16	**0.021**
**TNM stage** I II	525 1,061	487 793	38 268	**<0.001**

Bold values indicate significance of p value (p < 0.05).

**Table 2:** The relationships between adjuvant radiotherapy and clinicopathological characteristics.

Variables	No. of patients(n = 1,586)	Without ART(n = 1,405)	Receiving ART(n = 181)	P value
**Gender** Female Male	1,091 495	971 434	120 61	0.442
**Age** <70 >=70	673 913	584 821	89 92	0.051
**Race** Black White Others	202 1,211 173	174 1,080 151	28 131 22	0.384
**Grade** I II III+IV Unknown	385 759 338 104	352 665 289 99	33 94 49 5	**0.010**
**TNM stage** I II	525 1,061	506 899	19 162	**<0.001**

Bold values indicate significance of p value (p < 0.05).

### Independent Prognostic Indicators for Resected Gallbladder Adenocarcinoma

In the studied cohort, the median OS was 47 months (95% CI, 41–53 months). Concomitantly, the 1-, 3-, and 5-year OS rates were 80.9, 55.8, and 44.1%, respectively.

According to the univariate and multivariate analysis of OS, only age (p < 0.001; HR, 2.039; 95% CI, 1.718–2.420), tumor grade (p < 0.001; HR, 1.887; 95% CI, 1.530–2.370), and TNM stage (p < 0.001; HR, 1.417; 95% CI, 1.182–1.699) were shown to be independent prognostic risk factors (**Table 3**). Interestingly, ART and ACT were not found to have an independent prognostic effect in such this cohort analysis.

**Table 3:** Univariate and multivariate analysis of prognostic factors associated with overall survival in the overall cohort.

Variables	No. of patients (n = 1,586)	Overall survival			
		Univariate P value	Multivariate P value	Multivariate HR	95% CI
**Gender** Female Male	1,091 495	Ref. 0.368			
**Age** <70 >=70	673 913	Ref. **<0.001**	Ref. **<0.001**	2.039	1.718–2.420
**Race** Black White Others	202 1,211 173	Ref. 0.822 0.065			
**Grade** I II III+IV Unknown	385 759 338 104	Ref. **0.044** **<0.001** 0.915	Ref. 0.259 **<0.001** 0.889	1.127 1.887 1.027	0.916–1.386 1.503–2.370 0.706–1.494
**TNM stage** I II	525 1,061	Ref. **<0.001**	Ref. **<0.001**	1.417	1.182–1.699
**Radiotherapy** No Yes	1,405 181	Ref. 0.744			
**Chemotherapy** No Yes	1,280 306	Ref. 0.093			

Bold values indicate significance of p value (p < 0.05).

### Subgrouping Analyses for Age, Tumor Differentiation, and TNM Stage

**Figure 2:**
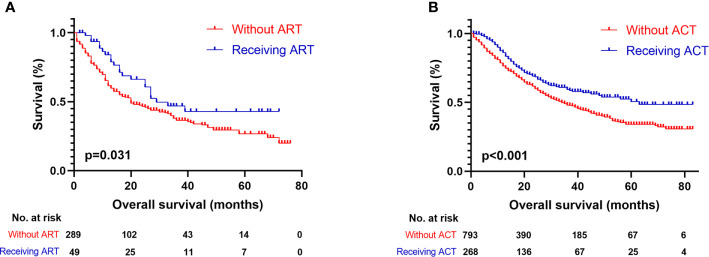
Kaplan-Meier survival curves for OS of patients with resected GBAC. **(A)** Survival comparison between patients with ART and without ART within tumor grade III/IV subgroup; **(B)** Survival comparison between patients with ACT and without ACT within tumor stage II subgroup.

When the patient cohort was subgrouped according to age, it was identified that tumor differentiation (p < 0.001; HR, 2.293; 95% CI, 1.474–3.569) and TNM stage (p < 0.001; HR, 2.090; 95% CI, 1.467–2.977) were the independent prognostic factors for patients under 70 years of age. However, for patients over 70 years of age, only tumor differentiation (p < 0.001; HR, 1.682; 95% CI, 1.291–2.192) was shown to be a significant risk factor (**Table 4**). The subgrouping of patients according to tumor differentiation (grades I+II and III+IV) revealed that advanced age (p < 0.001; HR, 2.281; 95% CI, 1.851–2.810) and higher TNM staging (p < 0.001; HR, 1.414; 95% CI, 1.140–1.753) were independent risk factors for grade I/II patients. However, regarding grade III/IV patients, ART (p = 0.049; HR, 0.639; 95% CI, 0.409–0.999) was shown to be a significant prognostic factor (**Table 5**). The median OS of grade III/IV patients receiving ART was 29 months (95%CI, 16–42 months), while those who did not receive ART were 20 months (95%CI, 14–26 months) (p = 0.031, **Figure 2A**). Therefore, ART can provide survival benefit for patients with poor or absent tumor differentiation.

**Table 4:** Univariate and multivariate analysis of prognostic factors associated with overall survival according to the subgrouping of age.

Variables	Overall survival					
	Age <70			Age >= 70		
	Univariate P value	Multivariate P value	HR, 95% CI	Univariate P value	Multivariate P value	HR, 95% CI
**Gender** Female Male	Ref. 0.829			Ref. 0.667		
**Race** Black White Others	Ref. **0.032** 0.007	Ref. 0.065 0.022	Ref. 0.717, 0.503–1.021 0.507, 0.284–0.906	Ref. 0.467 0.612		
**Grade** I II III+IV Unknown	Ref. **0.036** **<0.001** 0.070	Ref. 0.126 **<0.001** 0.012	Ref. 1.348, 0.919–1.978 2.293, 1.474–3.569 2.098, 1.174–3.748	Ref. 0.793 **<0.001** 0.133	Ref. 0.885 **<0.001** 0.165	Ref. 1.018, 0.796–1.303 1.682, 1.291–2.192 0.699, 0.421–1.159
**TNM stage** I II	Ref. **<0.001**	Ref. **<0.001**	Ref. 2.090, 1.467–2.977	Ref. **0.017**	Ref. 0.084	Ref. 1.205, 0.975–1.489
**Radiotherapy** No Yes	Ref. 0.199			Ref. 0.257		
**Chemotherapy** No Yes	Ref. 0.688			Ref. 0.580		

Bold values indicate significance of p value (p < 0.05).

**Table 5:** Univariate and multivariate analysis of prognostic factors associated with overall survival according to the subgrouping of tumor differentiation.

Variables	Overall survival					
	Grade I+II			Grade III+IV		
	Univariate P value	Multivariate P value	HR, 95% CI	Univariate P value	Multivariate P value	HR, 95% CI
**Gender** Female Male	Ref. 0.294			Ref. 0.528		
**Age** <70 >=70	Ref. **<0.001**	Ref. **<0.001**	Ref. 2.281, 1.851–2.810	Ref. **<0.001**	Ref. **<0.001**	Ref. 1.796, 1.280–2.521
**Race** Black White Others	Ref. 0.689 0.090			Ref. 0.602 0.769		
**TNM stage** I II	Ref. **<0.001**	Ref. **0.002**	Ref. 1.414, 1.140–1.753	Ref. 0.145		
**Radiotherapy** No Yes	Ref. 0.620			Ref. **0.035**	Ref. **0.049**	Ref. 0.639, 0.409–0.999
**Chemotherapy** No Yes	Ref. 0.290			Ref. 0.089		

Bold values indicate significance of p value (p < 0.05).

As showed in **Table 6**, age (p < 0.001; HR, 2.887; 95% CI, 2.033–4.100) and tumor differentiation (p = 0.006; HR, 1.909; 95% CI, 1.201–3.305) were considered as significant independent risk factors for patients with stage I tumors. On the other hand, for stage II tumor patients, ACT (p = 0.011; HR, 0.739; 95% CI, 0.586–0.932) was identified as the only factor involved in survival prediction. The median OS of stage II patients who received ACT was 63 months (95% CI, not available), which was significantly longer than those who did not receive it (34 months, 95% CI, 29–39 months) (p < 0.001, **Figure 2B**). Therefore, ACT should be suggested as an adjuvant strategy when the tumor invades beyond the muscularis.

**Table 6:** Univariate and multivariate analysis of prognostic factors associated with overall survival according to the subgrouping of TNM stage.

Variables	Overall survival					
	TNM stage I			TNM stage II		
	Univariate P value	Multivariate P value	HR, 95% CI	Univariate P value	Multivariate P value	HR, 95% CI
**Gender** Female Male	Ref. 0.116			Ref. 0.774		
**Age** <70 >=70	Ref. **<0.001**	Ref. **<0.001**	Ref. 2.887, 2.033–4.100	Ref. **<0.001**	Ref. **<0.001**	Ref. 1.685, 1.378–2.060
**Race** Black White Others	Ref. 0.185 0.046			Ref. 0.409 0.389		
**Grade** I II III+IV Unknown	Ref. 0.116 **<0.001** 0.933	Ref. 0.346 **0.006** 0.888	Ref. 1.203, 0.819–1.767 1.909, 1.201–3.035 0.959, 0.538–1.711	Ref. 0.424 **<0.001** 0.502	Ref. 0.460 **<0.001** 0.581	Ref. 1.097, 0.858–1.404 1.896, 1.457–2.468 1.150, 0.700-1.888
**Radiotherapy** No Yes	Ref. 0.182			Ref. 0.085		
**Chemotherapy** No Yes	Ref. 0.118			Ref. **<0.001**	Ref. **0.011**	Ref. 0.739, 0.586–0.932

Bold values indicate significance of p value (p < 0.05).

## Discussion

Currently, the choice of a standard adjuvant treatment for GBAC is still a controversial topic and needs to be further elucidated. It is worth mentioning that it has not yet been clarified whether the ACRT use can improve OS of the patients compared to those who have only undergone surgical resection. In a meta-analysis, Ren and colleagues (17) found that gallbladder carcinoma patients who received ART had a significantly better 5-year OS rate, especially those with lymph node-positive and margin-positive disease. In a recent study, Bohan et al. (18) reported that ACT did not improve any OS benefit for patients with stage I gallbladder cancer (p = 0.83), although it has been associated with improved OS in stage II patients. These data are similar to those obtained in our study. Mitin et al. (19) concluded that adjuvant therapy is associated with significantly improved 3-year OS after analyzing 5,029 T1-3N0-1-diagnosed gallbladder cancer patients from the National Cancer Data Base. On the other hand, the study provided by Mantripragada et al. (20) showed that adjuvant therapy had no effect on 3-year OS of early-stage patients, although they observed a 3-year OS benefit in locally advanced gallbladder cancer patients. A retrospective study showed that in resected T2-3N0M0 gallbladder cancer patients, the 5-year OS rate did not vary significantly between those who underwent ACRT and those who did not receive adjuvant treatments. Nevertheless, among T2-3N1-2M0 stage patients, those who received ACRT had a significantly higher 5-year OS rate compared to those who did not undergo any adjuvant treatment. These data indicate that ACRT can improve OS in lymph node-positive resected GBAC patients (12) and suggested that adjuvant therapies may be beneficial in certain patients of early-stage resected GBAC.

In our study, we found that ART and ACT may not provide significant survival benefit in early-stage GBAC patients who underwent surgical resection. However, ART and ACT have been associated with improved outcomes in certain subgroups. For tumor grade III/IV patients, ART (p = 0.049; HR, 0.639; 95% CI, 0.409–0.999) was identified as a significant prognostic factor. The median OS of grade III/IV patients who received ART (29 months, 95% CI, 16–42 months) was significantly higher than those who did not receive it (20 months, 95% CI, 14–26 months). This result suggests that ART can improve the survival of patients with poor or absent tumor differentiation. Moreover, among stage II tumor patients, ACT showed a significant longer median OS than those who did not receive it (63 months and 34 months, respectively). This result is consistent with a previous study performed by Chen and collaborators (21). The main difference between stage I and II tumors is that in the latter the depth of tumor invasion is greater and affects the muscularis mucosa. Thus, for patients with a tumor that invades beyond the muscularis, performing ACT should be suggested to improve patient survival.

This study has two main limitations that deserve comment. It is a retrospective analysis using the SEER database, which lacks many significant clinical characteristics and can provide some selection bias. In addition, these results were derived from data from Western countries and their general applicability needs to be further confirmed. However, it should be noted that this study investigated a relatively large sample size (n = 1,586), despite the low GBAC incidence. Thus, this study was able to conduct a comprehensive evaluation of the ACRT effects on early-stage GBAC patients who received radical surgical resection.

## Conclusion

Although the curative effects of ACRT following surgical resection in early-stage GBAC patients is a controversial issue, we found that patients with poor or absent tumor differentiation benefited from ART and tumor stage II patients (tumor invasion beyond muscularis) benefited from ACT. Our study provides supporting evidence for the clinical application of ACRT in early-stage GBAC patients. Further large-scale randomized controlled trials are needed to confirm our findings about the role of adjuvant therapies in early-stage resected GBAC patients.

## Data Availability Statement

Publicly available datasets were analyzed in this study. This data can be found here: Surveillance, Epidemiology, and End Results (SEER) database (https://seer.cancer.gov/).

## Ethics Statement

The studies involving human participants were reviewed and approved by the Ethics Committee of Naval Medical Center of PLA. Written informed consent for participation was not required for this study in accordance with the national legislation and the institutional requirements.

## Author Contributions

CL and YP conceptualized and designed the study, and acquired funding. CL, YW, JC, JX, and YP contributed to the methodology, provided the resources, and analyzed and interpreted the data. CL and YW wrote the manuscript. All authors read and approved the final manuscript. All authors contributed to the article and approved the submitted version.

## Conflict of Interest

The authors declare that the research was conducted in the absence of any commercial or financial relationships that could be construed as a potential conflict of interest.
